# Cytotoxic Activity and Antiproliferative Effects of Crude Skin Secretion from *Physalaemus nattereri* (Anura: Leptodactylidae) on *in vitro* Melanoma Cells

**DOI:** 10.3390/toxins7103989

**Published:** 2015-10-08

**Authors:** Andréa Cruz e Carvalho, César Augusto Prías Márquez, Ricardo Bentes Azevedo, Graziella Anselmo Joanitti, Osmindo Rodrigues Pires Júnior, Wagner Fontes, Mariana S. Castro

**Affiliations:** 1 Laboratory of Toxinology, Department of Physiological Sciences/IB, University of Brasília, Brasília/DF, CEP 70.910-900, Brazil; E-Mails: andreabiologa@gmail.com (A.C.E.C.); caprias@gmail.com (C.A.P.M.); osmindo@unb.br (O.R.P.J.); 2 Laboratory of Biochemistry and Protein Chemistry, Department of Cell Biology/IB, University of Brasília, Brasília/DF, CEP 70.910-900, Brazil; E-Mail: wagnerf@unb.br; 3 Department of Genetics and Morphology/IB, University of Brasília, Brasília/DF, CEP 70.910-900, Brazil; E-Mails: razevedo@unb.br (R.B.A); bygra1@gmail.com (G.A.J.); 4 Faculty of Ceilândia, University of Brasília, Ceilândia/DF, CEP 72.220-140, Brazil

**Keywords:** anurans, antitumoral activity, *Physalaemus nattereri*, skin secretion, melanoma, cytotoxicity

## Abstract

Anuran secretions are rich sources of bioactive molecules, including antimicrobial and antitumoral compounds. The aims of this study were to investigate the therapeutic potential of *Physalaemus nattereri* skin secretion against skin cancer cells, and to assess its cytotoxic action mechanisms on the murine melanoma cell line B16F10. Our results demonstrated that the crude secretion reduced the viability of B16F10 cells, causing changes in cell morphology (e.g., round shape and structure shrinkage), reduction in mitochondrial membrane potential, increase in phosphatidylserine exposure, and cell cycle arrest in S-phase. Together, these changes suggest that tumor cells die by apoptosis. This skin secretion was also subjected to chromatographic fractioning using RP-HPLC, and eluted fractions were assayed for antiproliferative and antibacterial activities. Three active fractions showed molecular mass components in a range compatible with peptides. Although the specific mechanisms causing the reduced cell viability and cytotoxicity after the treatment with crude secretion are still unknown, it may be considered that molecules, such as the peptides found in the secretion, are effective against B16F10 tumor cells. Considering the growing need for new anticancer drugs, data presented in this study strongly reinforce the validity of *P. nattereri* crude secretion as a rich source of new anticancer molecules.

## 1. Introduction

Melanoma is one of three common types of skin cancer which, in spite of many advances in treatment, is still considered one of the most devastating illnesses in the modern world. Melanoma occurs in melanocytes found in the skin and, among humans, affects mainly white individuals [1]. This type of skin cancer causes huge public health problems due to an increased annual incidence of 3%–8% [2]. This increase is mainly due to a rise in environmental exposure to ultraviolet rays, which can induce abnormalities in the genetic pathways of melanocytes. Melanoma treatment usually includes surgery, although some cases of advanced tumors require the use of systemic therapies with chemotherapeutic agents, such as dacarbazine, fotemustine, and temozolomide. Nevertheless, conventional treatments not always result in satisfactory outcomes, since cancer cells frequently develop resistance to these treatments or show low response rates [2,3]. Thus, the development of new drugs plays an important role in cancer treatment [4,5,6,7,8]. Although a number of new pharmacological agents alone or in combination has recently entered clinical trials, the discovery of new sources of biomolecules with anticancer potential is still a challenge for the pharmaceutical industry. Hence, amphibians can become an important source of these leader molecules. 

In general, the presence of mucous glands on the skin of anurans is associated with cutaneous respiration, reproduction, and water balance, whereas granular glands are related to the production of defense toxins [9]. Frogs and their skin secretion have been employed in therapeutic applications by many different cultures around the world [10,11,12]. This traditional knowledge led to the concept that the skin of anurans may be a rich source of new biologically active compounds. Research on toxic anuran secretions concerning the presence of biogenic amines, bufadienolids, alkaloids, and peptides was described two decades ago [13,14], and additional studies have been conducted in order to isolate and characterize active compounds from anuran skin. These studies have shown that dermal glands synthesize and excrete a variety of mammalian-like hormones, neuropeptides and kinins, as well as opioid peptides and cytolytic antimicrobial peptides. All of them are considered to be involved in the defense against mammal predation and/or microbial invasion/colonization of the frog skin. Some of these peptides can act as antiproliferative agents on cancer cells, and may even be non-toxic to normal cells [15]. 

Treatments based on antimicrobial peptides (AMPs) have emerged as new strategies for cancer therapy, since they have different major roles on the innate immunity of many organisms [16]. The use of these molecules is advantageous in many cases because they are more selective for the negatively charged membrane of cancer cells; show good tissue penetration due to their small size; act quickly; do not stimulate cell resistance; show synergism with classic drugs; have a broad spectrum of activity; are capable of destroying primary tumors; and prevent metastasis [17].

In *Physalaemus nattereri*, granular glands are found as a pair of black and round eye-like spots associated with deimatic behavior. The inguinal macroglands have small spherical granules with protein content, and have the function of discouraging the attacks of potential predators. If visual cues are insufficient and the predator persists in the attack, a poisonous secretion is released into its mouth [9]. Although this behavior was reported quite some time ago, little is known about the biochemical properties of the secretion. Hence, the aims of this study were to analyze the anticancer activity of crude skin secretion of the frog *Physalaemus nattereri* (Steindachner, 1863), and to study its cytotoxic mechanism on B16F10 murine melanoma cells.

## 2. Results

### 2.1. P. nattereri Crude Secretion Decreased Cell Viability in a Dose-Dependent Manner 

**Figure 1:**
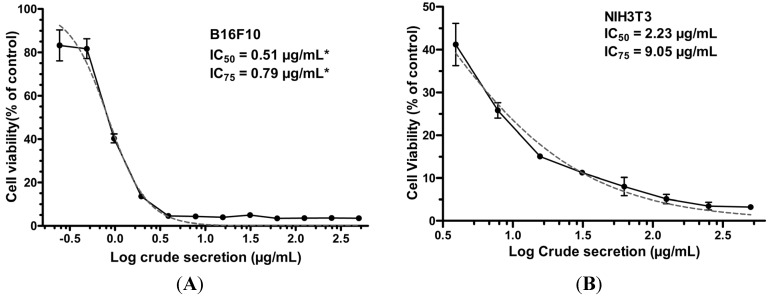
Effect of crude *P. nattereri* skin secretion on cell viability of melanoma (B16F10) (**A**) and normal fibroblasts (NIH3T3) (**B**) after a 24-h treatment with serial concentrations of the crude secretion. Cell viability was determined by the MTT assay. Data are expressed as means ± SD of experiments carried out in triplicate. * Showed values for B16F10 are from the confirmatory experiment based on data of first MTT assay.

Whole crude secretion of *P. nattereri* induced a dose-dependent reduction in cell viability in both melanoma cells and normal fibroblasts after a 24-h treatment (Figure 1). Nevertheless, the effect was more pronounced against melanoma cells, in which IC_50_ was approximately 4.4 times lower (0.51 μg/mL) than that required for normal fibroblasts (2.23 μg/mL). In order to investigate the mechanism of action of crude skin secretion on melanoma cells, subsequent experiments were performed using the IC_75_ dose (0.79 μg/mL), as described below.

### 2.2. Crude Skin Secretion Induced Changes in Cell Morphology 

**Figure 2:**
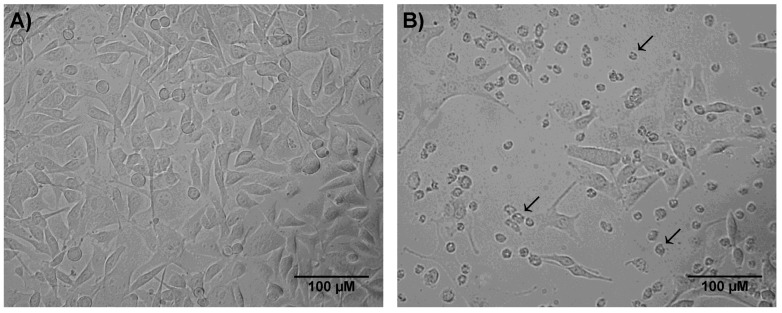
Morphological alterations in melanoma cells (B16F10) incubated with 0.79 μg/mL of *P. nattereri* crude skin secretion for 24 h, as assessed by contrast phase microscopy. (**A**) Control and (**B**) Treated cells. Bar = 100 μm, arrow = round-shaped and detached cells.

After 24 h of incubation with *P. nattereri* crude secretion, expressive morphological alterations of melanoma cells were observed (Figure 2), such as loss of cell prolongations, cell detachment, loss of spindle-shaped morphology and shrinkage.

### 2.3. Crude Skin Secretion Induced Slight Changes in Cell Size and Granularity

**Figure 3:**
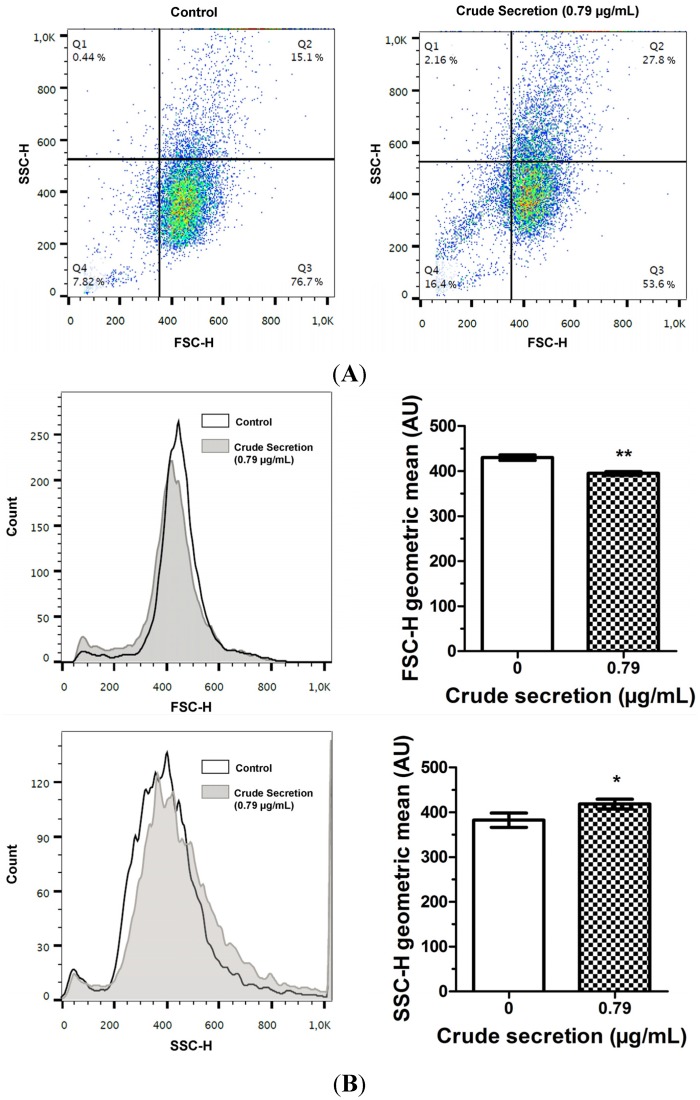
Cell morphology analysis by flow cytometry of B16F10 cells treated in triplicate for 24 h with 0 μg/mL (control) and 0.79 μg/mL crude skin secretion of *P. nattereri* (IC_75_). (**A**) Two-dimensional plot showing differences in size (FSC-H) and granularity (SSC-H) (**B**) Histogram and bar graphs of geometric mean showing differences for each parameter as mean ± SD. Total events: 10,000. Legend: * = *p* < 0.05, ** = *p* < 0.01.

Cell size (FSC-H) and granularity (SSC-H) were analyzed by flow cytometry (Becton, Dickinson and Company, Franklin Lakes, NJ, USA). Treatment with crude skin secretion induced alterations of these parameters indicating a general tendency to the reduction of cell size (Figure 3A, Q1 and Q4 and Figure 3B, FSC-H). In addition, a discreet increase in cell granularity was observed, as shown in Figure 3A (Q1 and Q2) and Figure 3B (SSC-H).

### 2.4. Crude Skin Secretion Caused Alterations in Melanoma Cell Plasma Membrane

**Figure 4:**
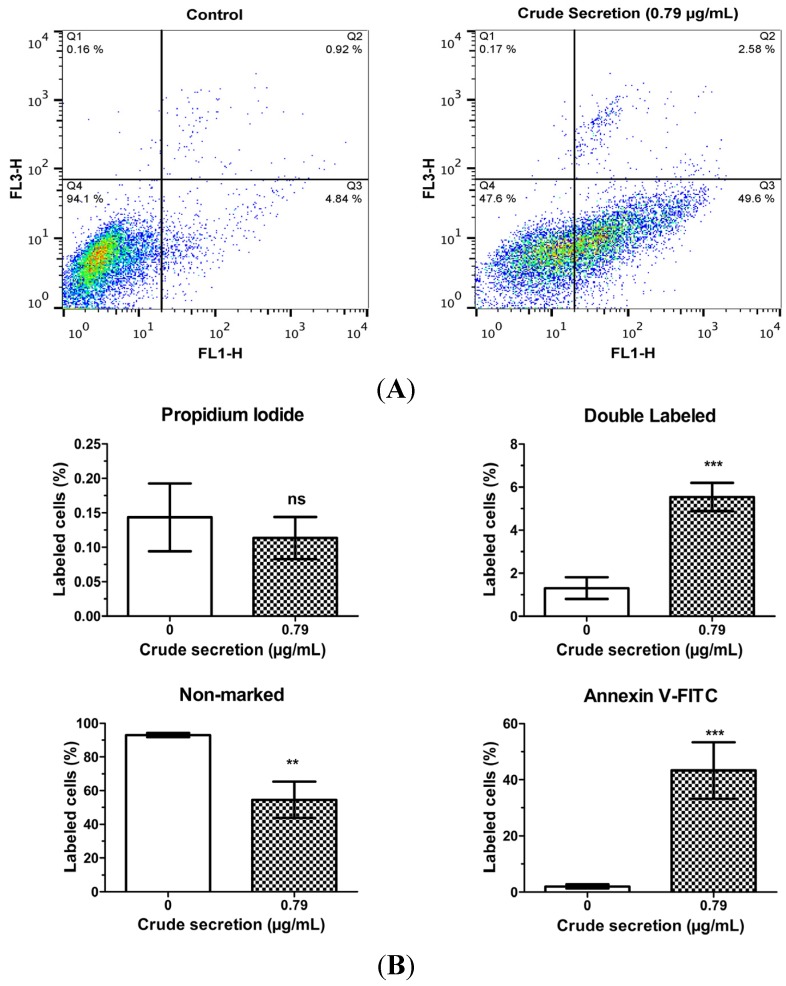
Effects of *P. nattereri* crude skin secretion on apoptosis and necrosis. These parameters were assessed by flow cytometric analysis in an experiment carried out in triplicate. (**A**) Annexin V/propidium iodide (PI) two-dimensional plots showing control (0 µg/mL) and treated (0.79 µg/mL) cells incubated for 24 h with *P. nattereri* crude skin secretion. The graphs shows four quadrants (Q1–Q4) representing cells marked only with PI (Q1), cells marked with both Annexin V and PI (Q2), cells marked only with Annexin V (Q3) and non-marked cells (Q4). (**B**) Bar graphs showing the percentage of cells in each quadrant, expressed as mean ± SD. Legend: ns = not significant, ** = *p* < 0.01, *** = *p* < 0.001.

Figure 4 shows that the treatment of melanoma cells with 0.79 μg/mL *P. nattereri* crude skin secretion for 24 h induced alterations in plasma membrane features regarding patterns of phosphatidylserine exposure (annexin V^+^ cells), and plasma membrane permeability (PI^+^ cells). An increase of 4.24% in the proportion of annexin V^+^ and PI^+^ cells was observed after treatment (1.31 ± 0.50% *vs.* 5.54 ± 0.66%; *p* < 0.001). Furthermore, there was a 41.26% increase in the number of cells labeled only with annexin V (2.05 ± 0.73% *vs.* 43.31 ± 10.02%; *p* < 0.001); and consequently, a 38.48% decrease (93.01 ± 1.20% *vs.* 54.53 ± 10.77%; *p* < 0.01) in the number of non-labeled cells. No significant differences were observed in the number of cells marked exclusively with PI (0.14 ± 0.49 *vs.* 0.11 ± 0.31; *p* > 0.05). The plasma membrane of untreated cells did not show expressive phosphatidylserine exposure or altered permeability with 94.1% of cell population showing no labeling for annexin V or PI markers.

### 2.5. Crude Skin Secretion Reduced Mitochondrial Membrane Potential of Melanoma Cells 

**Figure 5:**
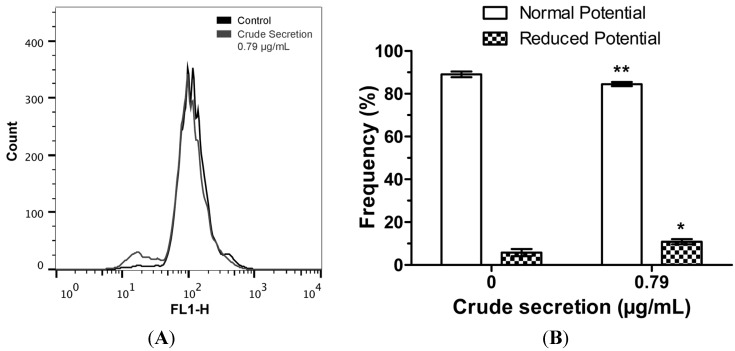
Mitochondria membrane potential in melanoma cells. This parameter was assessed with rhodamine 123 staining followed by flow cytometry. (**A**) Histogram of flow cytometry showing rhodamine 123 fluorescence in control cells and cells treated with 0.79 µg/mL crude skin secretion of *P. nattereri* for 24 h. (**B**) Bar graph showing differences in the frequency of cells with normal and altered mitochondrial membrane potential, expressed as mean ± SD. * = *p* < 0.05, ** = *p* < 0.01. Experiments were carried out in triplicate.

Analysis of functional features of mitochondria showed that *P. nattereri* crude skin secretion induced significant modifications in this organelle. Mitochondrial membrane potential was significantly reduced in the cells treated with 4.53% (*p* < 0.01), as evidenced by a decrease in the population of cells showing positive rhodamine 123 labeling (Figure 5). 

### 2.6. Crude Skin Secretion Induced Slight Changes in Cell Cycle Pattern of Melanoma Cells

**Figure 6:**
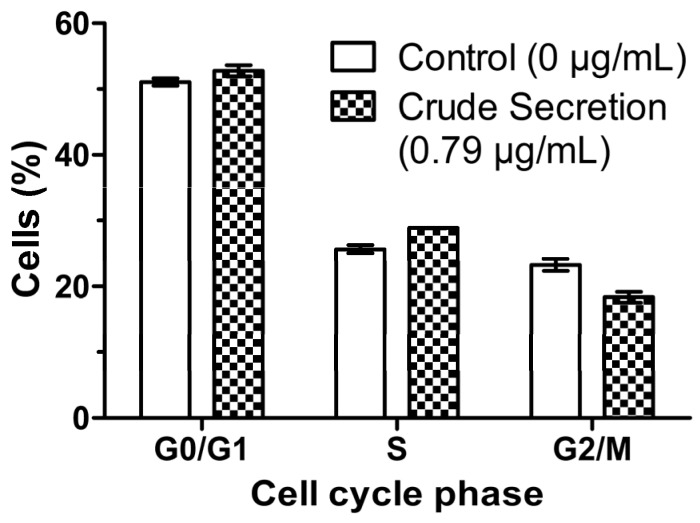
Effect of *P. nattereri* crude secretion on cell cycle phases of melanoma cells (B16F10) after 24 h of incubation. Cell cycle phases were analyzed by propidium iodide staining and flow cytometry. Data are expressed as the percentage of G1, S and G2/M cell cycle phases and represent the means ± SD of experiments carried out in triplicate. The control group corresponds to untreated cells.

In order to investigate the effects of crude skin secretion of *P. nattereri* on cell proliferation, melanoma cells were treated with 0.79 μg/mL of the secretion for 24 h and flow cytometric analysis was performed with propidium iodide staining. The results (Figure 6) showed slight differences in cell-cycle phases for untreated and treated cells. This difference was very discreet in G0/G1 phase (51.07% *vs.* 52.78%). Simultaneously, there was an increment of 3.24% (25.64% *vs.* 28.88%) in the proportion of cells in the S phase (Synthesis), and a decrease of 4.94% (23.29% *vs.* 18.35%) in the proportion of cells in the G2/M phase, showing that there was a tendency of cell cycle arrest in cells treated with the secretion. Statistical analysis of this experiment was not possible due to an insufficient number of events in one of the treatment replicates. 

### 2.7. Liquid Chromatographic Fractionation of P. nattereri Crude Skin Secretion, Antiproliferative, Hemolytic and Antibacterial Screenings, and Mass Spectrometry Analysis

**Figure 7:**
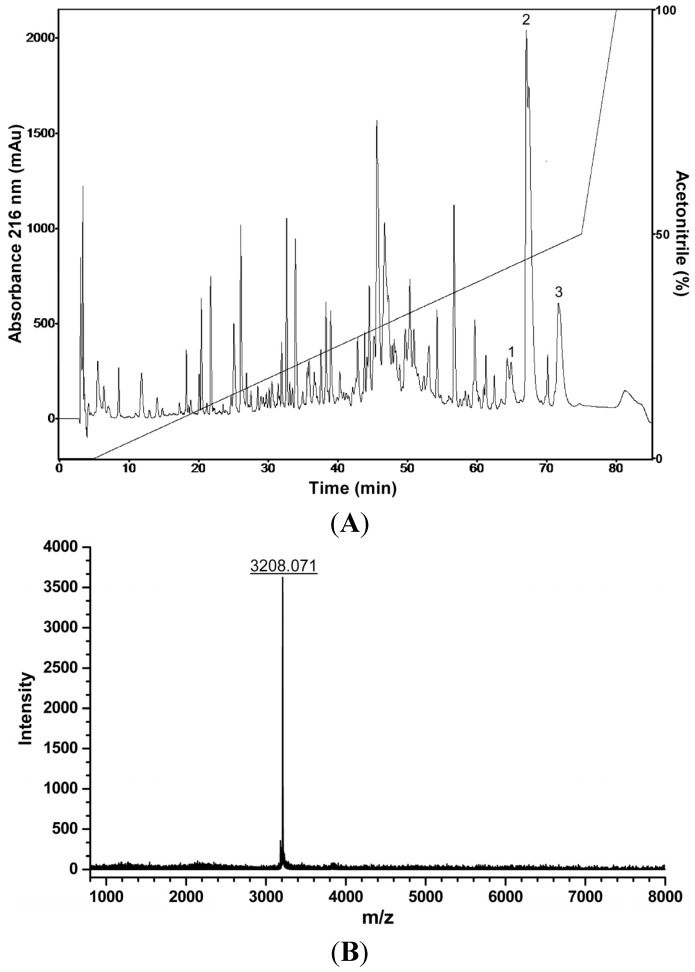
(**A**) Typical liquid chromatographic profile of *P. nattereri* crude skin secretion fractionation (RP-HPLC; Shim-Pack CLC-ODS, 6.0 × 150 mm; flow rate = 1 mL/min). The fractions indicated by numbers exhibited antiproliferative effects on melanoma cells, and antibacterial effects on *S. aureus*. The fraction indicated by number 2 was also active against *E. cloacae*. (**B**) Mass spectrum of the peptide purified from the fraction indicated by number 2 in the chromatogram, (Autoflex II TOF/TOF MS, Bruker Daltonics, Bremen, Germany; positive reflector ion mode).

Liquid chromatographic profile obtained with the fractionation of *P. nattereri* skin secretion by RP-HPLC resulted in approximately 140 eluted fractions (Figure 7A). All these fractions were assayed *in vitro* for their hemolytic activity on erythrocytes suspension, antiproliferative effects on melanoma cells (B16F10), as well as for their antibacterial activity against the Gram-positive bacteria *Staphylococcus aureus* (ATCC 25923) and the Gram-negative bacteria *Enterobacter cloacae* (ATCC 35030). Among the biologically active fractions, three hydrophobic fractions demonstrated to have deleterious effects on melanoma and *S. aureus* cells, one of them was also active against *E. cloacae* and no hemolytic effects were observed. Molecular mass analysis of these inhibitory fractions by MALDI-TOF MS revealed the presence of several compounds in the range of 2.4–3.8 kDa. Three antimicrobial peptides (with molecular masses of 3179; 3207 as shown in Figure 7B and 3141 Da determined by MALDI-TOF MS analysis) were purified from these chromatographic fractions. These peptides are active against pathogenic bacteria and melanoma cells in the µMolar range. The structural and biological characterization of these peptides is still in progress. 

## 3. Discussion

Several studies have validated the potential of anuran skin extracts and secretions as sources of anticancer agents. Studies conducted with a variety of samples have shown deleterious effects on gastric, colon, brain, lung, breast, bladder, and liver tumor cell lines, as well as on leukemia cell lines [18,19,20,21,22]. The antitumoral mechanisms of these molecules include regulation of the immune response, impairment of angiogenesis, reversal of multi-drug resistance, antiproliferative effects, and triggering of apoptosis in most cases [18,19,20,21,22,23,24,25]. In this context, skin secretions from different species (e.g. *Phyllomedusa hypocondrialis* and species of the genus *Rhinella*) have been explored; nevertheless, investigations of other anuran species as potential sources for new bioactive compounds remain to be unraveled. In the present work, the anticancer activity of crude skin secretion of the frog *Physalaemus nattereri* on B16F10 murine melanoma cells was evaluated. 

The MTT assay showed that skin secretion of *P. nattereri* reduced the viability of the murine cancer cell line B16F10 in a dose-dependent manner (Figure 1). After a 24-h treatment, a significant decrease in B16F10 cells incubated with 0.79 μg/mL of the secretion was observed compared to the control. Additionally, many of the cells had severe morphological alterations, suggesting that the crude secretion had a cytotoxic effect (Figure 2). This fact was also accompanied by discreet but significant changes in cell size and granularity, as observed in Figure 3. Morphological alterations, such as rounding and cell shrinkage are consistent with apoptotic cell death [26]. Indeed, pronounced alterations in cell shape and detachment from the surface were previously observed with other amphibian secretions [22]. Consistently, the annexin V/PI experiments performed with the crude skin secretion showed an increment in the number of cells marked positively for Annexin V, revealing cells in early stage apoptosis (Figure 4). Simultaneously, there was a discreet increase in double marked cells (Annexin V^+^/PI^+^), revealing the presence of cells undergoing late stage apoptosis or necrosis, which can occur as a consequence of cell membrane permeabilization provoked by components of the skin secretion, such as membrane-active peptides. 

Investigation of the cell death mechanism also revealed that B16F10 cells treated with crude secretion had their mitochondrial membrane potential altered (Figure 5), suggesting that these molecules are able to affect intracellular structures inducing mitochondrial membrane depolarization, which is also associated with apoptosis. Some AMPs interact with bacterial membranes, which share structural similarity with the external mitochondrial membrane of cells [27]. Apoptosis-inducing factors existent in mitochondrial intermembrane space may be released following external mitochondrial membrane disruption [28], and may be responsible for the initiation of the intrinsic apoptotic pathway, as was recently reported for a small wasp-derived tetrapeptide acting in B16F10-Nex2 cells [29].

It was observed a tendency of the skin secretion to inhibit cell proliferation of B16F10 cells, inducing the arrest of the S phase (Figure 6) and reducing the number of cells in the G2/M phase. Cell cycle progression is controlled by several Cyclin/CDK complexes [30]. Some studies have shown that besides inhibition of cyclin A/CDK2 kinase activity, p21 expression and inappropriate activation of E2F-1 genes may also be responsible for S phase arrest [31,32]. Further experiments will be performed to confirm the effects of *P. nattereri* skin secretion on cell cycle. That confirmation will be valuable for pharmaceutical purposes since cell cycle arrest may potentiate the cytotoxicity of cell cycle-disrupting chemotherapeutics.

Despite the cytotoxicity to NIH3T3 cells, the crude secretion should be more deeply studied as a possible alternative in melanoma therapy, since it presents an IC_50_ that is more than four times greater for NIH3T3 cells than for B16F10 cells. Strategies that increase selectivity could reduce side effects, for instance, by targeting the crude secretion towards the tumor using carriers such as liposomes or viruses, in such a way that it specifically binds to tumor cell membranes [33,34]. 

A few studies were conducted regarding the toxicity of *P. nattereri* secretion, and little is known about the molecular composition of this secretion. One study conducted by Lenzi-Mattos *et al.* [9] reports that the skin secretion of *P. nattereri* displays high toxicity against several possible vertebrate predators, exhibiting a LD_50_ of 27 µg, a low dose when compared to the venom of the viperid *Bothrops jararaca* (LD_50_ = 45 µg). It is known that amphibian alkaloids show a great diversity [35], and antiproliferative activities on tumor cells have been observed in toad skin secretions that are rich in certain alkaloids [22]. However, there is no current evidence supporting the efficiency of amphibian-derived alkaloids as antiproliferative or cytotoxic agents. Studies assessing the presence of alkaloids in the skin secretion of *P. nattereri* are required to suggest a possible alkaloid-driven mechanism. 

Cutaneous secretion of *P. nattereri* is rich in peptides as was demonstrated by the combination of liquid chromatography and off-line mass spectrometry analysis [36,37]. That fact was corroborated in our study by means of the fractionation of the crude secretion and additional chromatographic steps by RP-HPLC, and subsequent MALDI-TOF MS analysis, enabling the isolation of three peptides with antibacterial and *in vitro* antitumor activity on melanoma cells. Thus, these peptides present in this skin secretion may be responsible for at least some of the observed effects. 

Besides the antimicrobial role of the cationic peptides purified from amphibians, much evidence supports their use in cancer therapy, since AMPs present potent activity against tumor cells, have low immunogenicity and are small, reducing their cost of synthesis [38]. Certainly, a large number of AMPs have been isolated from amphibians and several of them are effective as anticancer agents. Ocellatin-P1 (pentadadactylin) isolated from the pepper frog *Leptodactylus labyrinthicus* (Leptodactylidae) possesses antiproliferative and cytotoxic activity against B16F10 but is also cytotoxic to normal human fibroblasts (FHN) at slightly higher concentrations [38]. Other peptides with anticancer properties include the antitumoral and angiostatic dermaseptins B2 and B3 isolated from *Phyllomedusa bicolor* (Hylidae) [15], the widely-active temporin-1CEa from *Rana chensinensis* (Ranidae) [39] and the high-potency, low-selective Hymenochirin-1Pa from *Pseudhymenochirus merlini* (Pipidae) [40]. Studies about the mechanism of action and the development of strategies capable of increasing the potency, selectiveness, and resistance to serum components can signify important progress in these therapeutic models [41].

In conclusion, we analyzed the skin secretion of the amphibian *Physalaemus nattereri* for antitumor activity. This secretion showed pronounced deleterious effects on B16F10 murine melanoma cell line. The exact mechanism that causes reduction in cell viability and cytotoxicity after treatment with the crude secretion is still unknown. Nevertheless, we found strong evidence of apoptosis-mediated cell death and further studies are necessary to validate the apparent arrest in cell cycle phase S. These results clearly reinforce the validity of crude secretion of *P. nattereri* as a rich source of new anticancer molecules, some of them related to the class of antimicrobial peptides. 

Subsequent steps include the isolation and biochemical characterization of peptidic molecules responsible for the observed effects on tumor cells in order to more deeply explore the mechanisms of action of *P. nattereri* skin secretion and their components. 

## 4. Experimental Section

### 4.1. Collection of Specimens and Skin Secretion

Adult specimens of *Physalaemus nattereri* were collected in Monte Alegre/State of Goiás (Brazil) and maintained in captivity at the University of Brasilia. The secretion from their cutaneous glands was obtained by mild electrical stimulation, and collected in a beaker by washing the skin surface with Milli-Q water (Merck Millipore, Billerica, MA, USA). The secretion was immediately lyophilized and then kept frozen (−20 °C) for subsequent use. No animals were harmed during these experiments, and they immediately reassumed their normal behavior.

### 4.2. Cell Treatment

B16F10 murine melanoma (ATCC CRL-6475) and NIH3T3 normal murine fibroblast (ATCC CRL-1658) cell lines were purchased from the Rio de Janeiro Cell Bank (NCE/UFRJ, Rio de Janeiro, Brazil). They were routinely maintained in culture flasks (TPP, Trasadingen, Switzerland) at 37 °C and 5% CO_2_ with Dulbecco’s modified Eagle’s medium (DMEM) supplemented with 10% (*v*/*v*) heat-inactivated fetal bovine serum (FBS, Invitrogen, Waltham, MA, USA), 100 IU/mL penicillin, and 100 µg/mL streptomycin.

### 4.3. Cell Viability Assay (MTT)

Cell viability was determined by the MTT (3-4,5-dimethylthiazol-2,5 biphenyl tetrazolium bromide) assay [42]. For this purpose, B16F10 and NIH3T3 cells were seeded on 96-well plates at a density of 7 × 10^3^ and 8 × 10^3^ cells/well respectively, and incubated overnight at 37 °C in 5% CO_2_. Then, the medium was replaced with fresh medium containing serially diluted *P. nattereri* crude skin secretion previously filtrated in 0.22 µm membranes (Millex GV, Merck Millipore, Billerica, MA, USA). The control group corresponded to untreated cells. After 24 h, the culture medium was replaced with MTT (0.5 mg/mL in culture media), and the cells were incubated for 3 h at 37 °C in 5% CO_2_. 

For purposes of confirming the inhibitory concentrations, before the flow cytometric assays, a second MTT assay was performed with B16F10 cells incubated in 12-well plates at a density of 5 × 10^4^ cells/well.

### 4.4. Investigation of the Mechanism of Action of P. nattereri Crude Skin Secretion on Melanoma Cells

In order to investigate the mechanism of action of *P. nattereri* crude skin secretion on melanoma cells, a concentration able to reduce cell viability by 75% was selected (IC_75_). This value was calculated based on the MTT viability curve using non-linear regression. The cells were seeded on 12-well plates, as described above, and after 24 h of incubation with the selected IC_75_ crude secretion concentration, they were harvested by trypsinization, centrifuged, and prepared for the assays described below. 

#### 4.4.1. Cell Morphology Analysis

The morphology of B16F10 cells was analyzed using an inverted contrast-phase microscope (Zeiss, Oberkochen, Germany) prior to trypsinization. The images were digitalized using a digital camera coupled to the microscope, and the software DinoCapture 2.0 Version 1.5.0. Parameters related to the size and granularity of treated cells were obtained by flow cytometry using FSC and SSC channels, respectively (Becton, Dickinson and Company, Franklin Lakes, NJ, USA). 

#### 4.4.2. Annexin V/Propidium Iodide Staining

Treated cells were resuspended in 100 µL of binding buffer containing 0.01 M Hepes (pH 7.4), 0.14 M NaCl and 2.5 mM CaCl_2_. Then, 5 µL Annexin V-FITC (BD Pharmingen™, Franklin Lakes, NJ, USA) and 10 µL propidium iodide (PI) (Sigma-Aldrich, St. Louis, MO, USA) were added and incubated for 15 min in the dark at 25 °C. Finally, 400 µL of binding buffer were added, and cells were immediately analyzed by flow cytometry. A total of 10,000 events were analyzed per sample.

#### 4.4.3. Mitochondrial Membrane Potential

Rhodamine 123 is a cationic fluorescent probe that has been shown to be selectively accumulated in the mitochondria, because of the negative transmembrane potential that these organelles maintain in living cells [43]. Thus, in order to investigate mitochondrial membrane potential, treated cells were washed two times with 500 μL of PBS. Then, 0.5 μL of Rhodamine 123 solution (5 mg/mL diluted in ethanol, Sigma, St. Louis, MO, USA) were added to each cell group and incubated for 15 min at room temperature. Next, the cells were washed two times with PBS and analyzed by flow cytometry. A total of 10,000 events were analyzed per sample.

#### 4.4.4. Cell Cycle Analysis

Propidium iodide (PI) is a fluorescent probe capable of binding and labeling DNA. Considering that PI fluorescence intensity is dependent on the DNA size, it is possible to precisely identify hypodiploid cells and cells at different stages of the cell cycle by flow cytometry [44]. To this purpose, treated cells were resuspended in 200 µL of 0.1% sodium citrate, 0.1% Triton X-100 and 20 μg/mL propidium iodide, phosphate buffer solution at pH 7.4 and then incubated for 30 min at room temperature, in the dark. The cell cycle was analyzed by a flow cytometry and a total of 10,000 events were analyzed per sample.

#### 4.4.5. Statistical Analysis

Statistical differences between control and treated cells were evaluated using unpaired Student’s *t*-test, at a significance level of 0.05, in Graph Pad Prism 5.03 (GraphPad Software, La Jolla, CA, USA). All values were expressed as means ± SD, corresponding to the analysis of three different experiments in each group. Values significantly different from the control at *p* < 0.05 are indicated in the figures with asterisks. All the experiments were carried out in triplicate.

### 4.5. Liquid Chromatographic Fractionation of P. nattereri Crude Skin Secretion, Antiproliferative, Hemolytic and Antibacterial Screenings, and Mass Spectrometry Analysis

Freeze-dried secretion (5.0 mg) was dissolved in 0.1% (*v*/*v*) TFA/water (220 μL), and subjected to RP-HPLC on a C_18_ column (Shim-Pack CLC-ODS, 6.0 I.D. × 150 mm, Shimadzu Corp., Kyoto, Japan) equilibrated with 0.1% (*v*/*v*) TFA/water (solvent A). After an initial 5-min wash with solvent A, elution was performed at a flow rate of 1 mL/min with a 0%–50% linear gradient of acetonitrile containing 0.1% (*v*/*v*) TFA (solvent B) for 70 min, then from 50% to 100% of solvent B for 5 min and a final 5-min wash with 100% solvent B. Absorbance was monitored at 216 nm. All fractions were manually collected, lyophilized, and stored for subsequent analysis. Individual chromatographic fractions obtained after five chromatographic runs were pooled and assayed to evaluate their antiproliferative effects on B16F10 cells, according to the method described in Section 2.1, and also to assess their antibacterial activity against *S. aureus* and *E. cloacae*, as described in Castro *et al.* [45]. These active fractions were individually submitted to additional chromatographic steps using a C_18_ column (Vydac 218TP54, 4.6 I.D. × 250 mm, W. R. Grace and Company, Columbia, MD, USA) and three purified peptides were obtained. These peptides were subjected to mass spectrometric analysis by MALDI-TOF MS using an Autoflex II TOF/TOF mass spectrometer (Bruker Daltonics, Bremen, Germany) in the 000–4000 Da range using the reflector positive mode. α-Cyano-4-hydroxycinnamic acid (HCCA) was used as the matrix. External calibration was performed using MH^+^ ions of angiotensin II (*m*/*z* = 1046.54), angiotensin I (*m*/*z* = 1296.68), substance P (*m*/*z* = 1347.74), bombesin (*m*/*z* = 1619.82), and adrenocorticotropic hormone 1–17 (*m/z* = 2093.09), adrenocorticotropic hormone 18–39 (*m*/*z* = 2465.20). 
